# The Extent of Irradiation-Induced Long-Term Visceral Organ Damage Depends on Cranial/Brain Exposure

**DOI:** 10.1371/journal.pone.0122900

**Published:** 2015-04-02

**Authors:** François-Xavier Boittin, Josiane Denis, Jean-François Mayol, Patrick Martigne, Florent Raffin, David Coulon, Nancy Grenier, Michel Drouet, Francis Hérodin

**Affiliations:** Department of Radiobiology, IRBA (Institut de Recherche Biomédicale des Armées), Brétigny-sur-Orge, France; University of California, Los Angeles, UNITED STATES

## Abstract

In case of high-dose radiation exposure, mechanisms controlling late visceral organ damage are still not completely understood and may involve the central nervous system. To investigate the influence of cranial/brain irradiation on late visceral organ damage in case of high-dose exposure, Wistar rats were irradiated at 12 Gy, with either the head and fore limbs or the two hind limbs protected behind a lead wall (head- and hind limbs-protected respectively), which allows long-term survival thanks to bone marrow protection. Although hind limbs- and head-protected irradiated rats exhibited similar hematopoietic and spleen reconstitution, a late body weight loss was observed in hind limbs-protected rats only. Histological analysis performed at this time revealed that late damages to liver, kidney and ileum were attenuated in rats with head exposed when compared to animals whose head was protected. Plasma measurements of inflammation biomarkers (haptoglobin and the chemokine CXCL1) suggest that the attenuated organ damage in hind limbs-protected rats may be in part related to reduced acute and chronic inflammation. Altogether our results demonstrate the influence of cranial/brain exposure in the onset of organ damage.

## Introduction

In addition to acute radiation syndrome, exposure to high-dose radiation induces long-term damage in visceral organs such as lung, liver, kidney or gastro-intestinal tract. These delayed radiation effects may even lead to multiple organ dysfunction syndrome (MODS) and life-threatening multiple organ failure (MOF) [1]. Radiation-induced MODS/MOF (RI-MODS/MOF) observed in irradiated patients appears to share similarities with the MODS/MOF occurring in shock, sepsis, pancreatitis or thermal burns, with progressive and sequential loss of function of vital organs [1,2].

Irradiation-induced late organ damage has long been considered as resulting mainly from the loss of stem cells, leading to altered repopulation or abnormal tissue remodelling [1]. However, numerous studies provide other hypotheses to explain the onset of organ damage after irradiation. Radiation-induced organ damage is thought to be related to an excessive systemic inflammatory response syndrome (SIRS). Acute inflammation with neutrophil granulocyte infiltration into tissues such as lung or brain has been observed just after irradiation [3–5]. Moreover, chronic inflammation, with repetitive increased expression of pro-inflammatory cytokines has been observed into tissues [6–8]. Both acute and chronic inflammation may be involved in radiation-induced late organ damage, as anti-inflammatory treatments have been demonstrated to be beneficial regarding late organ damage/dysfunction [9–12]. In addition to pro-inflammatory mediator release, excessive production of reactive oxygen species by inflammatory cells and damages to the endothelial monolayer in vessels may also contribute to irradiation-induced late organ damage [13–16].

All these mechanisms leading to organ damage in case of irradiation may be even more complex if interactions between damaged organs and altered systemic circulation occur. Moreover, the central nervous system may also play a role in the development of late organ damage [17]. Increased cytokine levels and inflammation in brain structures have indeed been reported following irradiation [18]. However, if the radiosensitivity of the central nervous system has been demonstrated, its role in the development of long-term organ damage and RI-MODS/MOF remains to be investigated [17].

In order to investigate the influence of cranial/brain irradiation in the development of late organ damage, Wistar rats were irradiated at 12 Gy, with either the head and fore limbs or the two hind limbs protected behind a lead wall. These two irradiation configurations allow long-term survival of rats thanks to bone marrow protection. We provide here histological evidences that late damages to visceral organs are attenuated when rat head has been exposed to irradiation, which may be in part related to reduced acute and chronic inflammation. Overall our results strongly suggest that the central nervous system influences the onset of visceral organ damage in case of high-dose exposure.

## Material and Methods

### Animals and ethics statements

Wistar rats (male, specific pathogen free) were purchased from JANVIER CERJ (Le Genest Saint Isle, France). They were housed in a controlled 12 h light/dark cycle and fed a standard solid diet and water *ad libitum*. 12 weeks old rats (~370 g) were used in all experiments. This study was carried out in strict accordance with the European legislation for the care of laboratory animals. Protocols were approved by the French Army Ethics Committee (protocol n^o^ 2012/01.09), in accordance with European rules and regulations (Directive 2010/63/UE, 22/09/2010). Irradiation of animals was performed under anaesthesia. All efforts were made to minimize suffering.

### Irradiation

**Fig 1:**
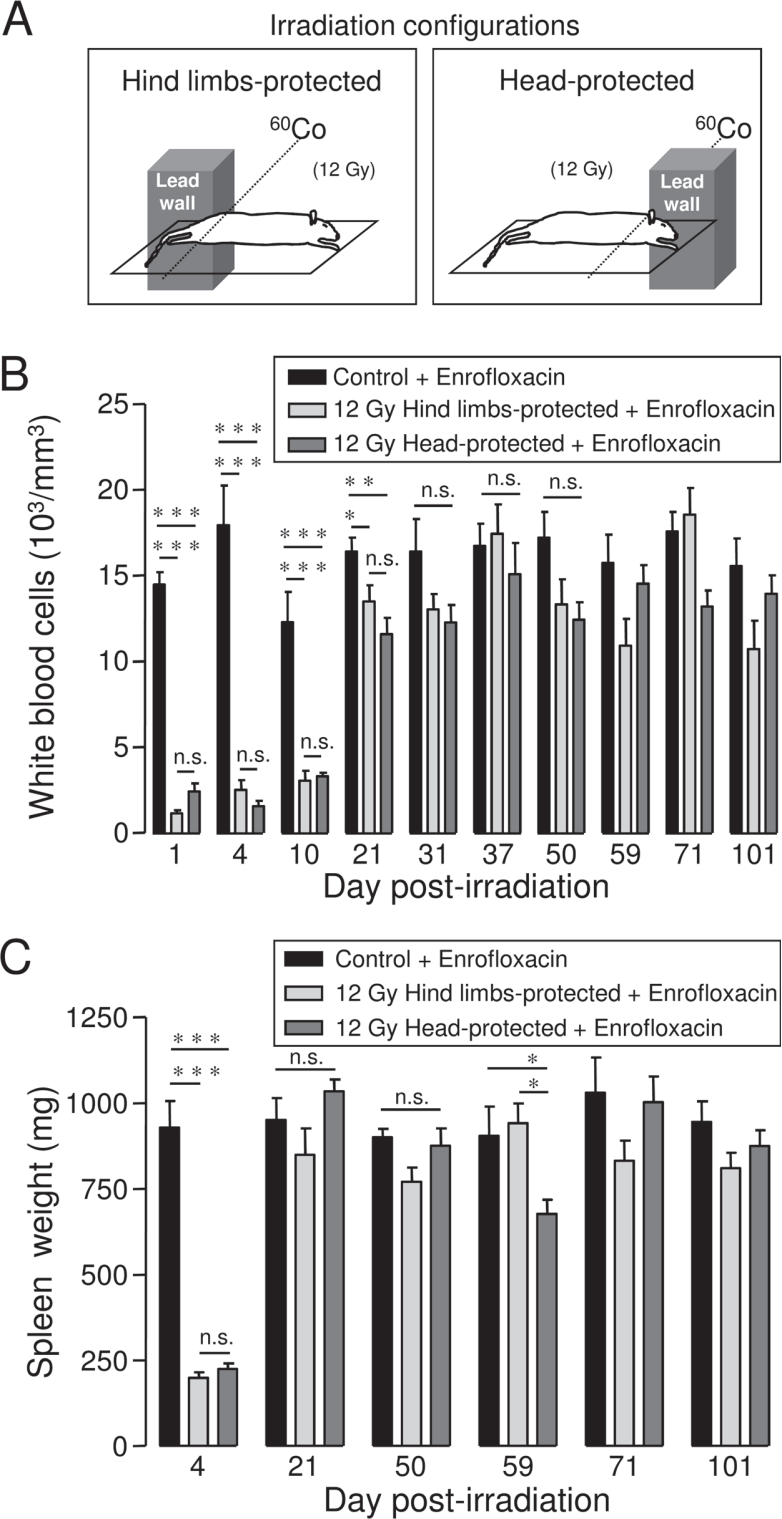
Irradiation configurations, white blood cells counts and spleen weight in highly-irradiated rats with bone marrow protection. A: Irradiation configurations. Rats were irradiated at 12 Gy gamma ray dose using à ^60^Co gamma source, with either the two hind limbs (“hind limbs-protected”) or the head and the two fore limbs (“head-protected”) protected behind a lead wall. Detailed description of irradiation configurations is included in the material and methods section. B: Kinetic of white blood cells counts in hind limbs- and head-protected rats irradiated at 12 Gy. White blood cells counts were performed from whole blood collected by retro-orbital puncture just before euthanasia. For each day post-irradiation (1, 4, 10, 21, 31, 37, 50, 59, 71, 101), data represent average values obtained from 4–5 hind limbs-protected rats, 4–5 head-protected rats and 5 age-matched control rats from the same batch. C: Kinetic of spleen weight recovery in hind limbs- and head-protected rats. Rat spleens were weighed 4, 21, 50, 59, 71 and 101 days after irradiation. For each day after irradiation, data represent average values obtained from 4–5 hind limbs-protected rats, 4–5 head-protected rats and 5 age-matched control rats. Control and irradiated rats received Enrofloxacin treatment. (*** (p<0.001); ** (p<0.01); * (p<0.05); n.s.: not significant).

All rats (control and irradiated) were anaesthetized using intraperitoneal injection of ketamine (IMALGENE 1000, 120 mg/kg) and acepromazine (CALMIVET, 0.05 ml/rat). Once anaesthetized, rats were placed four by four, each one over the other on the superimposed shelves (space between shelves: 12 cm) of a custom-made Plexiglas building, with stretched limbs immobilized using adhesive tape. Rats were then irradiated at a dose of 12 Gy using a ^60^Cobalt gamma source (dose rate 0.65 Gy/min). Accuracy of radiation doses was controlled using both an ionization chamber (PTW probe) and thermo-luminescent dosimeters. As shown in Fig. 1A, rats were either irradiated with the two hind limbs or the head and the two fore limbs protected behind a lead wall (10 cm thickness). Behind the lead wall, dose reduction calculated using thermoluminescent dosimeters placed on fully shielded parts of animals (hind limbs) was ~97%. In the case of hind limbs-protected rats, bone marrow from femurs, tibias and hind foots was protected, while bone marrow from the skull, mandible, fore foots, humerus, radius, ulna, cervical vertebras and part of thoracic vertebras was protected in the case of head-protected rats. According to a study performed in rats, we estimated that the volume of bone marrow protected in our hind limbs- and head-protected models represents ~20–25% of total bone marrow [19].

For our irradiation configurations (Fig. 1A), a dose distribution along rat body together with the dose distribution in the field of penumbra at the edge of the lead wall has been calculated using the RayXpert software, a Monte-Carlo 3D calculation. This software allows simulating photons and electrons based on GEANT physics (www.geant4.org/geant4). Thus, the volume behind the lead shield was modelled by one hundred sixty two cubes of 1 cm^3^ each. The dose delivered in each of these cubes was calculated. Dose distribution was found to be uniform along the part of rat body fully exposed to the ^60^Co source for both hind limbs- and head-protected rats. Calculation of dose distribution in the cubic detectors (1 cm^3^) placed at the edge and behind the lead wall revealed that the field of penumbra with intermediate dose distribution was very narrow at the edge of the lead wall. From 0 to 1 cm behind the lead wall, the calculated dose reduction was found to be ∼91% for our two irradiation configurations. From 1 to 2 cm and 2 to 3cm behind the lead wall, the calculated dose reduction were found to be respectively ∼96% and ∼97% for our setup, in accordance with values measured using thermo-luminescent dosimeters.

### Antibiotic support and animal care

Control and irradiated rats received fluoroquinolone antibiotic support. Enrofloxacin (BAYTRIL) was added to drinking water from day 1 to 22 post-irradiation (133 mg/l), corresponding to a daily dose of ∼5 mg/kg.

### Kinetic analysis of blood parameters and plasma haptoglobin, albumin, corticosterone and inflammatory mediators

In order to measure the evolution of blood parameters or plasma level of proteins and hormones, 12 Gy-irradiated rats with hind limbs or head protection and age-matched control animals were euthanized in the morning at 1, 4, 10, 21, 31, 37, 50, 59, 71 and 101 days after irradiation.

Before euthanasia, blood was collected under general anaesthesia (using Isofluran) by retro-orbital puncture (with EDTA as anti-coagulant). Blood cell counts were performed using a Pentra 120 analyser (ABX, Montpellier, France). Haptoglobin and albumin levels were measured in rat plasma using a Hitachi 912 automatic analyser. Plasma corticosterone levels were measured in the plasma of rats using corticosterone enzyme immunoassays (EIA) kits (Enzo Life Sciences), according to the manufacturer instructions. The sensivity of the corticosterone immunoassay is 26.99 pg/ml.

Cytokines (TNF-ɑ, Interleukin-1 (IL-1) and Interleukin-6 (IL-6)) and Cytokine-Induced Neutrophil Chemoattractant-1 (CINC1/CXCL1) plasma levels were measured using Quantikine ELISA kits (R&D Systems), according to the manufacturer instructions. The sensivity of the CXCL1 immunoassay is 1.1 pg/ml.

### Histological analysis of organ damage

Histological analysis was performed 64 days after 12 Gy-irradiation of rats with either hind limbs or head protection. All irradiated rats received antibiotic support from day 1 to 22 after irradiation (Enrofloxacin). Age-matched rats from the same batch were used as controls. To investigate potential effects of the three weeks Enrofloxacin treatment on histological analysis, control animals were treated or not with Enrofloxacin.

Rats were euthanized by decapitation after being anaesthetized using Isofluran. Liver, kidney ileum and brain were quickly removed and fixed in Formaldehyde. Samples were then rinsed, dehydrated and embedded in paraffin. Longitudinal sections (0.5 μm thickness) were cut using a microtom, placed on slides and stained with Hematoxylin, Eosin and Safran (HES). A Leica DMLB microscope, connected to a digital camera associated with Leica LAS image acquisition software (version 3), was used to capture all images. Histological qualitative analysis was performed to assess the severity of lesions observed in organs of irradiated rats.

For liver, kidney and ileum, scores of severity were determined, ranging from 0 to 2 or 3 depending on the parameters analysed. Parameters used for analysis of lung and kidney lesions are presented below, together with the scales used to assess the severity of lesions.

### Liver

-Hepatocytes (from 0 (normal) to 3 (major alteration)).
-Hepatocyte rows (from 0 (normal) to 2 (absent)).
-Steatosis (from 0 (absent) to 3 (major)).
-Sinusoids (0 (normal), 1 (dilated), 2 (dilated and damaged), 3 (ischemic)).
-Fibrosis (from 0 (absent) to 2 (major)).
-Macrophages (from 0 (absent) to 2 (many)).
-Lymphocytes (from 0 (absent) to 2 (many)).
-Necrosis (from 0 (absent) to 2 (major)).

### Kidney

-Glomeruli (from 0 (normal) to 2 (atrophic or dysmorphic)).
-Tubular structure (from 0 (normal) to 3 (major alteration)).
-Epithelial cells (from 0 (normal) to 3 (major alteration)).
-Fibrin deposition (from 0 (absence) to 3 (significant)).
-Vascular dilation (from 0 (absence) to 2 (ischemia)).
-Inflammatory cells (from 0 (absence) to 3 (many)).
-Fibrosis (from 0 (absence) to 2 (significant)).
-Necrosis (from 0 (absence) to 3 (major)).
-Medulla (from 0 (normal) to 3 (major alteration)).

### Ileum

-Overall structure (from 0 (normal) to 2 (major modification)).
-Epithelium:
  -Enterocytes (from 0 (normal) to 2 (major alteration)).
  -Caliciform cells (from 0 (few) to 2 (many)).
  -Brush border (from 0 (normal) to 2 (major erosion)).
  -Necrosis (from 0 (absence) to 2 (major)).
-Chorion:
  -Thickness (from 0 (normal) to 2 (thick)).
  -Capillaries (0 (normal), 1 (congestion)).
  -Lymphocytes (0 (present), 1 (many)).
  -Eosinophiles (0 (present), 1 (many)).
-Crypts:
  -Structure (0 (normal), 1 (alteration)).
  -Mitosis (from 0 (many) to 2 (rare)).
  -Inflammation (0 (absent), 1 (mild)).
-Muscular mucosa (from 0 (normal) to 2 (major alteration)).
-Under-mucosa (from 0 (normal) to 2 (major alteration)).
-Smooth muscle layers (0 (normal), 1 (alteration)).

Then a semi-quantitative assessment of histological lesions was performed for each organ by adding histological scores for each parameter analysed. Cumulative histological scores of severity are presented in Tables 1, 2 and 3 for liver, kidney and ileum. For ileum, measurements of villus length/width and villus number/mm were performed from HES images using Image J software.

**Table 1:** Histological scores of severity for livers collected 64 days after irradiation from 12 Gy-irradiated rats with hind limbs or head protection treated with Enrofloxacin and control rats treated or not with Enrofloxacin.

**Parameters**	**C**	**C + E**	**12 Gy HL-p + E**	**12 Gy H-p + E**
Hepatocytes	1,67	2	2.67	2.33
Hepatocyte rows	0	0	0.67	1.67
Steatosis	0	0	0	0
Sinusoids	0.67	0.67	0.33	1.33
Fibrosis	0	0	0	0.67
Macrophages	0.67	0	1	1
Lymphocytes	1	1	1	1
Necrosis	0	0	1	1.33
**Global Histological Score**	**4 ±0.58**	**3.67 ±0.33**	**6.67 ±0.33 (** * **)**	**9.33 ±0.88 (** *** **,** ^§^ **)**

*Rat Groups*: *C*: Control; *C + E*: Control + Enrofloxacin; *12 Gy HL-p + E*: 12 Gy Hind limbs-protected + Enrofloxacin; *12 Gy H-p + E*: 12 Gy Head-protected + Enrofloxacin. For each parameter and for each rat group, values represent the average of scores obtained in three animals. Global histological scores represent average values (± SEM) of cumulated histological scores obtained from three rats for each group

* (p<0.05);

*** (p<0.001): significant versus control + Enrofloxacin;

§ (p<0.05): significant versus 12 Gy hind limbs-protected + Enrofloxacin).

**Table 2:** Histological scores of severity for kidneys collected 64 days after irradiation from 12 Gy-irradiated rats with hind limbs or head protection treated with Enrofloxacin and control rats treated or not with Enrofloxacin.

**Parameters**	**C**	**C + E**	**12 Gy HL-p + E**	**12 Gy H-p + E**
Glomeruli	0	0	1	1.67
Tubular structure	0	0.33	1.33	2.67
Epithelial cells	0.33	0.33	1.67	2.67
Fibrin deposition	0.33	0.67	1	1
Vascular dilation	0.67	0	1	1.33
Inflammatory cells	0.33	0	1	1.33
Fibrosis	0	0	0	0.33
Necrosis	1	0.33	1	2
Medulla	0	0	0	1
**Global Histological Score**	**2.67 ±0.33**	**1.67 ±0.88**	**8 ±0.58 (** ** **)**	**14 ±1.73 (** *** **,** ^§§^ **)**

*Rat Groups*: *C*: Control; *C + E*: Control + Enrofloxacin; *12 Gy HL-p + E*: 12 Gy Hind limbs-protected + Enrofloxacin; *12 Gy H-p + E*: 12 Gy Head-protected + Enrofloxacin. For each parameter and for each rat group, values represent the average of scores obtained in three animals. Global histological scores represent average values (± SEM) of cumulated histological scores obtained from three rats for each group

** (p<0.01);

*** (p<0.001): significant versus control + Enrofloxacin;

§§ (p<0.01): significant versus 12 Gy Hind limbs-protected + Enrofloxacin).

**Table 3:** Histological scores of severity for Ileums collected 64 days after irradiation from 12 Gy-irradiated rats with hind limbs or head protection treated with Enrofloxacin and control rats treated or not with Enrofloxacin.

**Parameters**	**C**	**C + E**	**12 Gy HL-p + E**	**12 Gy H-p + E**
Structure	0	0.33	1.67	1.67
Enterocytes	0.33	0.33	1	1.67
Caliciform cells	0	0	1.33	1
Brush border	0	0.67	1	1.67
Necrosis	0	0	0.67	1
Chorion thickness	0.33	0.33	0.67	1.33
Chorion capillaries	0	0.33	0.33	0.67
Chorion lymphocytes	0	0	0.67	1
Chorion eosinophiles	0	0	0.33	0.67
Crypt structure	0.67	0.33	0.33	1
Crypt mitosis	0	0.67	0.33	1.67
Crypt inflammation	0	0	0	1
Muscular mucosa	0	0	0.33	0.33
Under-mucosa	0	0.33	1.67	0.33
Smooth muscle layers	0	0.67	1	0.67
**Global Histological Score**	**1.33 ±0.33**	**4 ±1.53**	**11.33 ±0.33 (** ** **)**	**15.67 ±1.45 (** *** **,** ^§^ **)**

*Rat Groups*: *C*: Control; *C + E*: Control + Enrofloxacin; *12 Gy HL-p + E*: 12 Gy Hind limbs-protected + Enrofloxacin; *12 Gy H-p + E*: 12 Gy Head-protected + Enrofloxacin. For each parameter and for each rat group, values represent the average of scores obtained in three animals. Global histological scores represent average values (± SEM) of cumulated histological scores obtained from three rats for each group

** (p<0.01);

*** (p<0.001): significant versus control + Enrofloxacin;

§ (p<0.05): significant versus 12 Gy Hind limbs-protected + Enrofloxacin

### Statistical analysis

Results are expressed as mean ± SEM. The number of rats used is indicated in the figure legends. One way ANOVA was performed to compare the mean values between groups. *P* values of >0.05 were considered as significant.

## Results

Rats were irradiated at 12 Gy-gamma ray dose with bone marrow protection achieved by protecting either both hind limbs or the head and fore limbs behind a lead wall, as represented in Fig. 1A. About 20–25% of total bone marrow was protected using these irradiation configurations [19]. Irradiated and control rats received antibiotic support using Enrofloxacin added in drinking water from day 1 to 22 post-irradiation. Bone marrow protection combined with antibiotic support allowed long-term survival of rats. Indeed, without any bone marrow protection, 12 Gy-irradiated rats died within 20 days.

### Hematopoietic recovery and spleen reconstitution in 12 Gy-irradiated rats with bone marrow protection (hind limbs or head protection)

Analysis of white blood cell counts was performed in hind limbs- and head-protected rats irradiated at 12 Gy, to investigate the kinetics of hematopoietic recovery. Results from Fig. 1B indicate near complete loss of white blood cells 1 day post-irradiation in both groups of irradiated rat. Bone marrow protection then allowed white blood cells counts to recover toward values close to those of age-matched control rats around 30 days post-irradiation for both hind limbs- and head-protected rats irradiated at 12 Gy. No significant differences were found between hind limbs- or head-protected rats regarding the kinetics of hematopoietic recovery.

Spleens of both 12 Gy-irradiated and age-matched control rats were weighed 4, 21, 50, 59, 71 and 101 days post-irradiation. Fig. 1C shows that average spleen weight was strongly decreased on day 4 after irradiation for both hind limbs- and head-protected rats, when compared to age-matched controls, as shown previously [20]. Average spleen weight of both hind limbs- and head-protected irradiated rats was not significantly different from age-matched controls 21 days after irradiation. Therefore no significant differences in spleen recovery were found between hind limbs- and head-protected rats. However, after the recovery phase observed in the two groups of irradiated rats, spleen weight was found to be decreased 59 days after irradiation in head-protected rats only.

### Evolution of body weight in 12 Gy-irradiated rats with hind limbs or head protection

**Fig 2:**
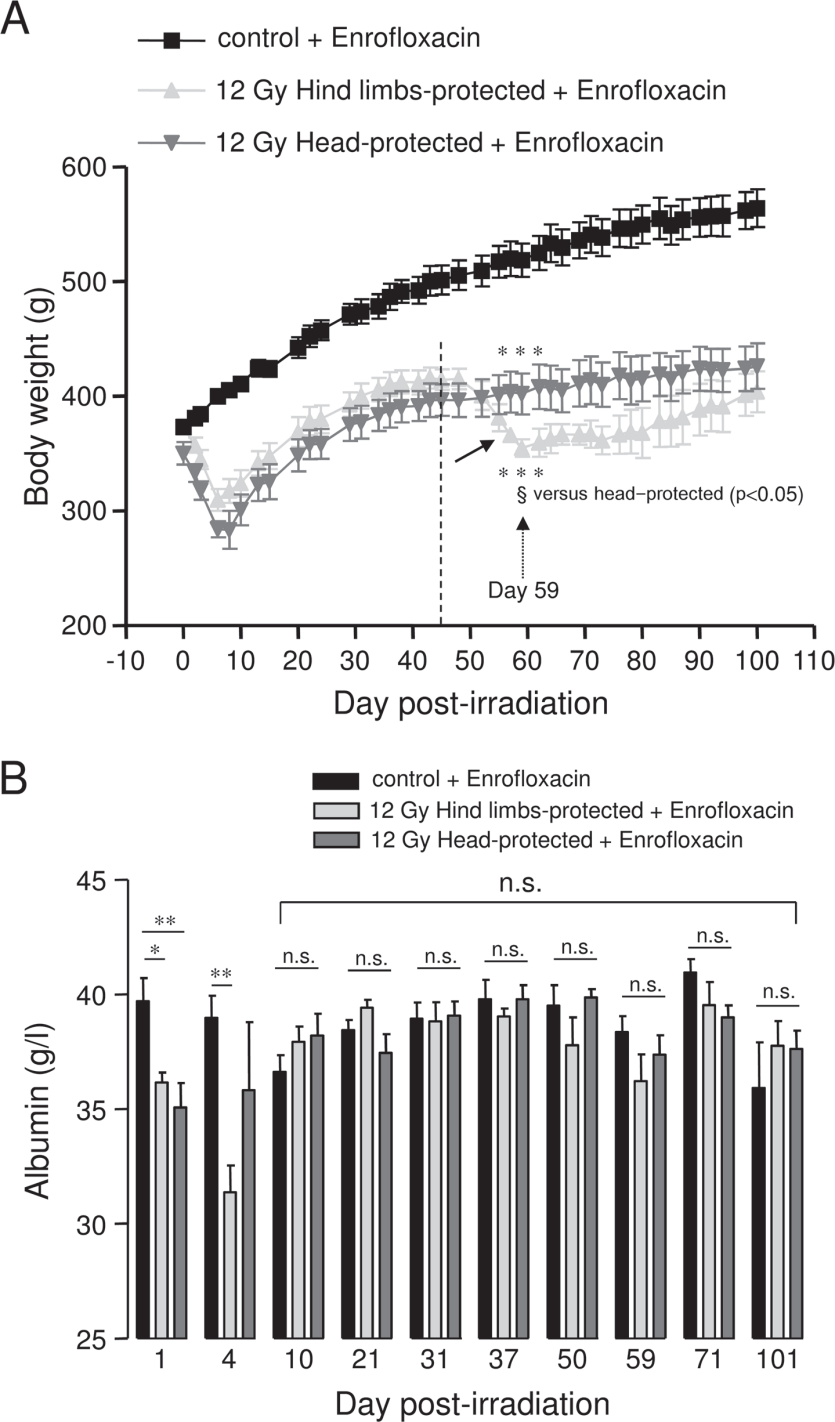
Evolution of body weight and plasma albumin levels in 12 Gy-irradiated rats with hind limbs or head protection. A: Body weight evolution for controls (5 rats) and 12 Gy-irradiated rats with either hind limbs (5 rats) or head protected (4 rats). All rats received antibiotic support (Enrofloxacin). Measurements of body weight were performed every 2–3 days. The arrow on the graph indicates late average body weight loss for hind limbs-protected rats. The evolution of body weight is represented for rat groups euthanized 101 days after irradiation. The same body weight evolution was observed for all groups of hind limbs-, head-protected and control rats euthanized earlier after irradiation. (*** (p0.001) significant versus control + enrofloxacin; § (p<0.05): significant versus head-protected). B: Kinetic analysis of plasma albumin levels in 12 Gy-irradiated rats with hind limbs or head protection. Plasma albumin levels were measured using a Hitachi 912 automatic analyser. For each day post-irradiation (1, 4, 10, 21, 31, 37, 50, 59, 71, 101), data represent average values obtained from the plasma of 4–5 hind limbs-protected rats, 4–5 head-protected rats and 5 age-matched control rats from the same batch. All animals were treated with Enrofloxacin. (** (p<0.01); * (p<0.05); n.s.: not significant).

Control and irradiated rats were weighed every 2–3 days. As shown in Fig. 2A, both hind limbs- and head-protected rats exhibited severe and rapid body weight loss until 8 days post-irradiation, due to acute radiation syndrome. This was followed by a recovery phase for both groups of irradiated rats. However, a second late phase of body weight loss, starting about 45 days post-irradiation, was observed in hind limbs-protected rats only (arrow in Fig. 2A). At 59 days post-irradiation, average body weight of hind limbs-protected rats was significantly reduced when compared to head-protected animals, while average body weight of both hind limbs- and head-protected irradiated rats was significantly reduced when compared to age-matched controls. Age-matched control rats receiving antibiotic support exhibited continuous body weight increase throughout the time course of experiments, as can be expected from rats fed *ad libitum* (Fig. 2A).

### Kinetic analysis of plasma albumin in 12 Gy-irradiated rats with hind limbs or head protection

Kinetic analysis of plasma albumin was performed in 12 Gy-irradiated rats with hind limbs or head protection and age-matched controls. Plasma albumin levels were found to be significantly decreased soon after irradiation (day 1) in both hind limbs- and head-protected rats (Fig. 2B), in accordance with previous report [21]. However, between 10 and 101 days post-irradiation, plasma albumin levels of both groups of 12 Gy-irradiated rats were not different from those of age-matched control animals (Fig. 2B). This indicates that from 10 days post-irradiation, irradiated animals were not dehydrated and also that they did not suffer from severe under-nutrition [22,23].

### Histological analysis of visceral organ damage in 12 Gy-irradiated rats with hind limbs or head protection 64 days post-irradiation

HES staining was used to investigate irradiation-induced organ damage 64 days post-irradiation, at the time of the second late phase of body weight loss observed in hind limbs-protected rats (Fig. 2A).

**Fig 3:**
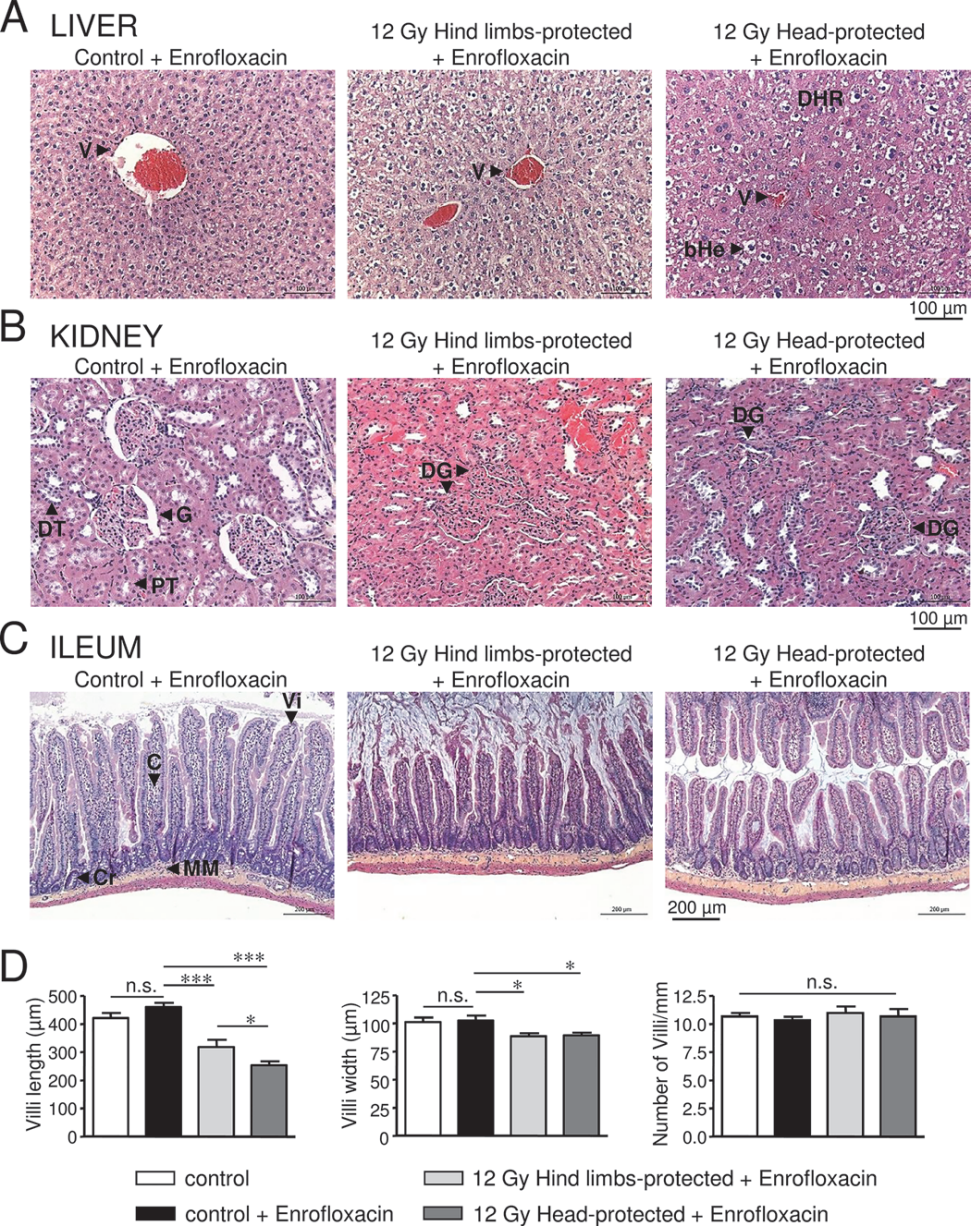
Histological analysis of liver, kidney and ileum damages in 12 Gy-irradiated rats with hind limbs or head protection 64 days post-irradiation. A: Representative pictures of liver sections stained with HES for control rats and 12 Gy-irradiated rats with either hind limbs or head protection. (V: Vessel, DHR: Disorganised Hepatocyte Rows, bHe: ballooned Hepatocytes). Control and irradiated rats were treated with Enrofloxacin. B: Representative pictures of kidney sections stained with HES for control rats and 12 Gy-irradiated rats with either hind limbs or head protection. (G: Glomerulus, DG: Dysmorphic Glomerulus, PT: Proximal Tubule, DT: Distal Tubule). Control and irradiated rats were treated with Enrofloxacin. C: Representative pictures of ileum sections stained with HES for control rats and 12 Gy-irradiated rats with either hind limbs or head protection. (Vi: Villus, C: Chorion, Cr: Crypt, MM: Muscular Mucosa). Control and irradiated rats were treated with Enrofloxacin. D: Measurements of villus length/width and villus number/mm performed using ileum HES staining images. Data on left and middle graphs represent the average of 12 measurements (from 3 rats in each condition). Analysis was performed from 3 control rats, 3 control rats treated with Enrofloxacin, 3 hind limbs-protected and 3 head-protected rats irradiated at 12 Gy. Both groups of 12 Gy-Irradiated rats received Enrofloxacin treatment. (*** (p<0.001); * (p<0.05); n.s.: not significant).

**Fig 4:**
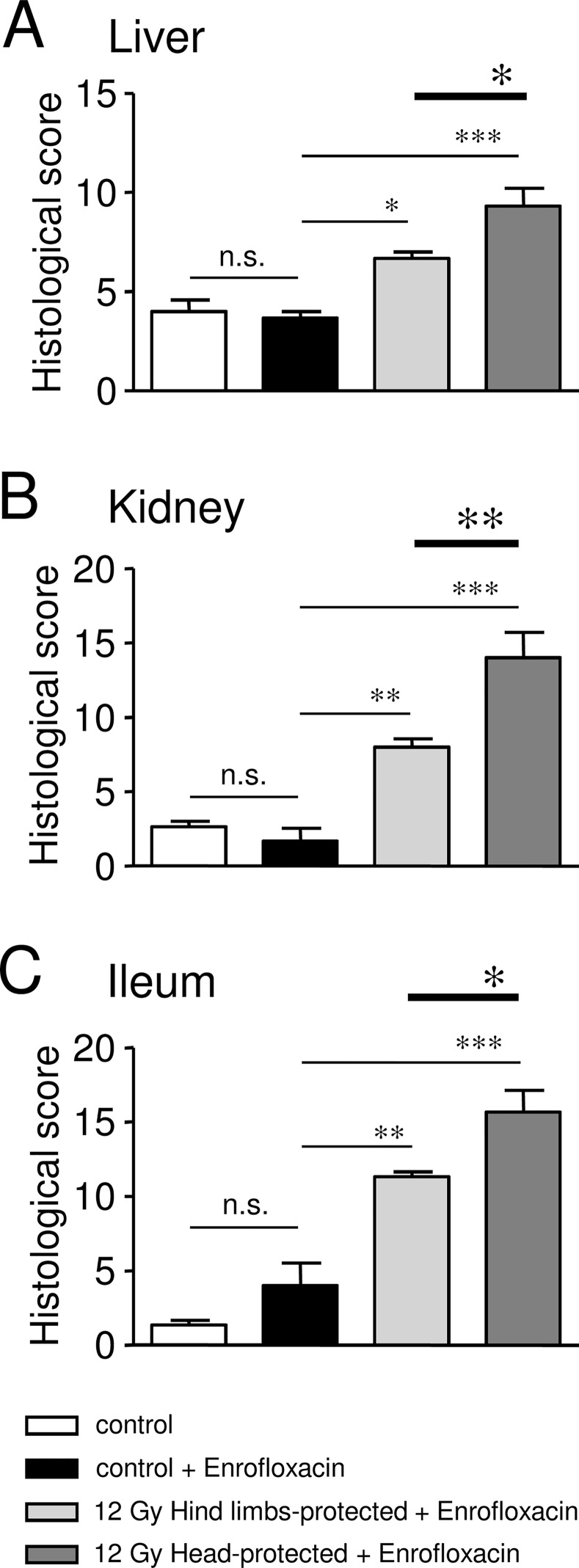
Histological scores of severity obtained for liver, kidney and ileum in control and 12 Gy-irradiated rats with hind limbs or head protection 64 days post-irradiation. A: Bar chart showing cumulative histological scores for liver in control (treated or not with Enrofloxacin) and 12 Gy-irradiated rats with hind limbs or head protection (treated with Enrofloxacin). B: Bar chart showing cumulative histological scores for kidney in control (treated or not with Enrofloxacin) and 12 Gy-irradiated rats with hind limbs or head protection (treated with Enrofloxacin). C: Bar chart showing cumulative histological scores for kidney in control (treated or not with Enrofloxacin) and 12 Gy-irradiated rats with hind limbs or head protection (treated with Enrofloxacin). Data were obtained from 3 controls, 3 controls treated with Enrofloxacin, 3 hind limbs-protected rats irradiated at 12 Gy and 3 head-protected rats irradiated at 12 Gy. (*** (p<0.001); ** (p<0.01); * (p<0.05); n.s.: not significant).

Livers of 12 Gy-irradiated rats with hind limbs or head protection exhibited major structural alterations regarding hepatocyte rows, which appeared disorganised. Sinusoidal dilation (with dilated endothelial cells), necrotic areas and ballooned hepatocytes were also observed in irradiated rats (Fig. 3A). However, hepatocyte alterations may result from treatments used for HES staining, as control rats also exhibited hepatocyte alterations. Disorganisation of hepatocyte rows, sinusoidal dilation and necrosis were increased in head-protected rats when compared to hind limb-protected ones, as confirmed by the increased histological scores for each of these parameters (Fig. 3A, Table 1)). Moreover fibrosis was only observed in the liver of head-protected rats (Table 1). In accordance with qualitative histological analysis, semi-quantitative analysis indicates that the global histological score of head-protected rats regarding liver lesions is significantly increased when compared to hind limbs-protected rats (Fig. 4A, Table 1).

Kidneys of 12 Gy-irradiated rats exhibited marked disorganised structure of superficial cortex, with altered glomeruli structure (atrophic, dysmorphic, with reduced Bowman space and dilated capillaries), altered tubular structure, altered tubular epithelial cells, vascular dilation/congestion (with altered endothelial cells), moderate fibrin deposition, increased number of inflammatory cells and necrotic areas (Fig. 3B). All these alterations were found to be exacerbated in head-protected when compared to hind limbs-protected rats, as confirmed by increased histological scores of severity regarding these parameters (Table 2). Moreover damages were found to expand up to the medulla in the case of head-protected rats (Table 2). Therefore the global histological score of head-protected rats regarding kidney alterations is significantly increased when compared to hind limbs-protected rats (Fig. 4B, Table 2).

The ileum overall structure was strongly affected by irradiation for both hind limbs- and head-protected rats irradiated at 12 Gy. As shown in Fig. 3C and D, villus length and width were significantly reduced in both irradiated rat groups when compared to control rats, but the villus length decrease was more pronounced in head-protected rats compared to their hind limbs counterpart. Similar alterations of villi have already been described after irradiation [24,25]. However in both irradiated rat groups, the number of villi per mm was not found to be different from control rats (Fig. 3C and D), indicating that only villus length and width were affected 64 days post-irradiation. Therefore the absorption surface of ileum is reduced in both hind limbs- and head-protected irradiated rats. Careful examination of ileum epithelial structure of irradiated rats revealed altered enterocyte ultrastructure, brush border erosion, increased caliciform cell number and necrotic areas when compared to controls. Most of these alterations were found to be exacerbated in head-protected when compared to hind limbs-protected rats, as indicated by increased histological scores regarding these parameters (Table 3). Chorion structure was also found to be impaired in both groups of irradiated rats, with increased thickness, damaged capillaries (congestion) and increased number of lymphocytes and eosinophiles. All these alterations were also found to be exacerbated in head-protected when compared to hind limbs-protected rats (Table 3). Moreover, crypt mitosis was altered in head-protected rats and crypt inflammation was only observed in these animals (Table 3). Alterations of muscular mucosa and under-mucosa were also observed in irradiated rats (Table 3). Overall, irradiation-induced ileum alterations were found to be increased in head-protected rats when compared to hind limbs-protected rats. As a consequence, the global histological score of head-protected rats regarding ileum lesions is significantly more severe when compared to hind limbs-protected rats (Fig. 4C, Table 3).

Brains of both hind limbs- and head-protected rats were also compared to controls using HES staining. Studies concerned potential thrombosis, amyloid deposition, edema, tumor, inflammation or congestion, but no obvious histological alterations were found in the brain of both irradiated rat groups (not shown).

### Kinetic analysis of plasma haptoglobin levels in 12 Gy-irradiated rats with hind limbs or head protection

**Fig 5:**
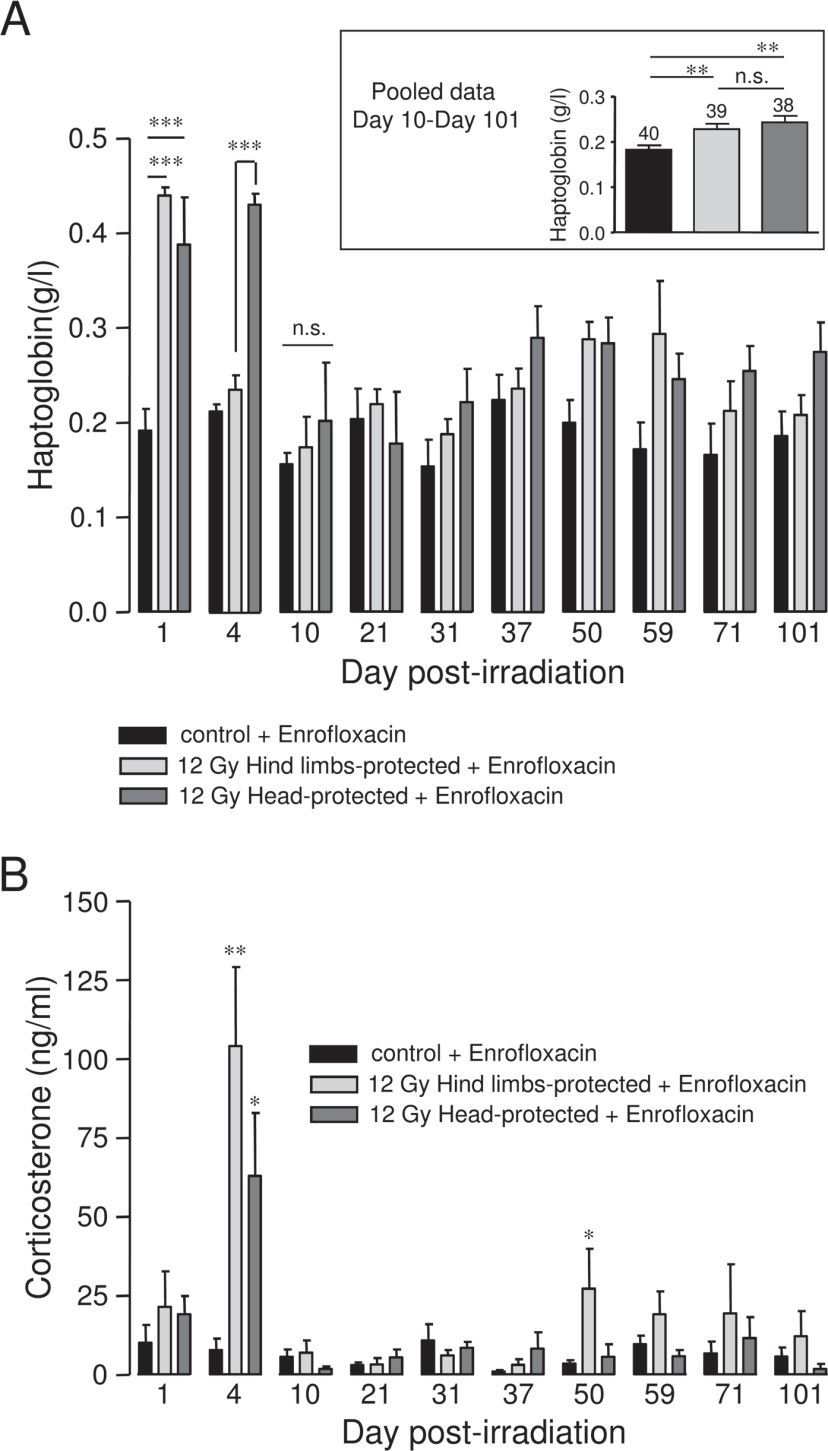
Kinetic analysis of plasma haptoglobin and corticosterone levels in 12 Gy-irradiated rats with hind limbs or head protection. A: Plasma haptoglobin level was measured using a Hitachi 912 automatic analyser. For each day post-irradiation (1, 4, 10, 21, 31, 37, 50, 59, 71, 101), data represent average values obtained from the plasma of 4–5 hind limbs-protected rats, 4–5 head-protected rats and 5 age-matched control rats from the same batch. All animals were treated with Enrofloxacin. Insert shows average values of haptoglobin plasma levels obtained by pooling data from 10 to 101 days post-irradiation. The number of rats used is indicated on the bar chart. B: Plasma corticosterone levels were measured using enzyme immunoassays (EIA) kits. For each day post-irradiation (1, 4, 10, 21, 31, 37, 50, 59, 71, 101), data represent average values obtained from the plasma of hind limbs-protected rats, head-protected rats and age-matched control rats from the same batch. All animals were treated with Enrofloxacin. (*** (p<0.001); ** (p<0.01); * (p<0.05); n.s.: not significant).

Kinetic analysis of plasma haptoglobin levels was performed in control and 12 Gy-irradiated rats with hind limbs or head protection. Haptoglobin is an acute phase protein of inflammatory reaction and a reliable marker of inflammation [26,27]. Plasma haptoglobin level was found to be greatly and significantly increased in both hind limbs- and head-protected rats 1 day post-irradiation, when compared to controls (Fig. 5A). However, while haptoglobin level returned to a value close to control in the case of hind limbs-protected rats 4 days post-irradiation, it remained significantly increased in head-protected rats, suggesting prolonged acute inflammatory reaction in head-protected rats (Fig. 5A). In both hind limbs- and head-protected rats, plasma haptoglobin levels returned to control levels 10 days post-irradiation. Insert in Fig. 5A shows average plasma haptoglobin values for control and irradiated rats during the late phase of irradiation. These results indicate that between 10 and 101 days post-irradiation, average plasma haptoglobin levels were significantly increased in both groups of irradiated rats when compared to controls. Therefore this indicates that late chronic inflammation developed in both groups of irradiated animals.

### Kinetic analysis of plasma corticosterone levels in 12 Gy-irradiated rats with hind limbs or head protection

Kinetic analysis of plasma corticosterone levels was performed in control and 12 Gy-irradiated rats with hind limbs or head protection using enzyme immunoassays (EIA) kits. Plasma corticosterone levels of control animals were ~10–15 ng/ml, in the range reported in previous studies [28,29]. As shown in Fig. 5B, plasma corticosterone levels were significantly increased in both hind limbs- and head-protected rats when compared to controls 4 days post-irradiation, with higher average value found in hind limbs-protected rats (Fig. 5B). A late significant increase in plasma corticosterone was only measured in hind limbs-protected rats (50 days post-irradiation, Fig. 5B). Interestingly, the late increase in plasma corticosterone levels observed in hind-limbs-protected rats occurred at the time these animals experienced body weight loss (Fig. 2A).

### Kinetic analysis of inflammatory mediator levels in the plasma of 12 Gy-irradiated rats with hind limbs or head protection

**Fig 6:**
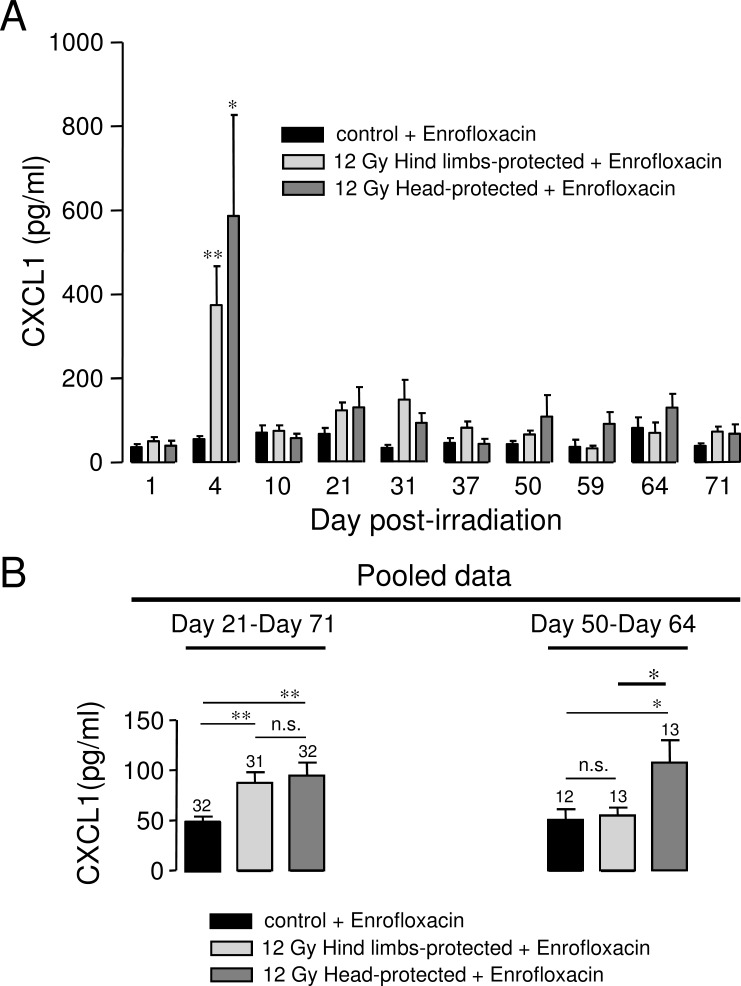
Kinetic analysis of plasma CXCL1 levels in 12 Gy-irradiated rats with hind limbs or head protection. A: Plasma CXCL1 levels were measured by ELISA. For each day post-irradiation (1, 4, 10, 21, 31, 37, 50, 59, 64, 71), data represent average values obtained from the plasma of 3–5 hind limbs-protected rats, 3–5 head-protected rats and 3–5 age-matched control rats from the same batch. Data from Day 64 correspond to the animals used for histology analysis. All animals were treated with Enrofloxacin. B: Average values of CXCL1 plasma levels obtained by pooling data from several days after irradiation for controls and irradiated rats with hind limbs or head protection (left panel: from 21 to 71 days post-irradiation; right panel: from 50 to 64 days post-irradiation). The number of rats used is indicated on bar charts. (** (p<0.01); * (p<0.05); n.s.: not significant).

Plasma levels of Cytokine-Induced Neutrophil Chemoattractant-1 (CINC1/CXCL1) and pro-inflammatory cytokines (TNF-α, IL-1 and IL-6) were measured by ELISA. Plasma levels of pro-inflammatory cytokines (TNF-α, IL-1 and IL-6) were not found to be different in irradiated animals when compared to controls between 1 and 101 days post-irradiation in our rat models (data not shown). Very transient increases in pro-inflammatory cytokines have been described a few hours following irradiation [3,5,18]. Therefore, plasma increases in pro-inflammatory cytokines may have occurred before our first measurement (24 hours post-irradiation). In contrast, we found significant plasma increases in Cytokine-Induced Neutrophil Chemoattractant-1 (CINC1/CXCL1) during both the acute and late phase after irradiation in our irradiated rat models (Fig. 6). CXCL1 belongs to the chemokine family. It is a major neutrophil chemoattractant and represents a reliable marker of inflammation [30,31]. Data from Fig. 6A indicates that CXCL1 plasma levels were strongly increased in both groups of irradiated rats 4 days post-irradiation, with the highest individual values found in rats with head protection. These results are in accordance with recent reports showing early increase in CXCL1 after irradiation [3,32]. However, increases in CXCL1 were also observed in the plasma of hind limbs- and head-protected irradiated rats in the late phase of irradiation. Data from Fig. 6B represents average values of CXCL1 plasma levels in control and irradiated animals between 21 and 71 days after irradiation (left panel) and at the very late phase after irradiation (between 50 and 64 days after irradiation, right panel). Results from Fig. 6B (left panel) indicate that CXCL1 plasma levels were significantly increased in both hind limbs- and head-protected rats between 21 and 71 days post-irradiation. In contrast, CXCL1 plasma levels were only found to be increased in head-protected rats at the very late phase of irradiation (between 50 and 64 days post-irradiation, Fig. 6B, right panel).

## Discussion

Using hind limbs- and head-protected rat models, we investigated here the influence of cranial/brain exposure in the development of long-term organ damage in case of high dose irradiation. Late damage in organs such as liver, kidney and ileum was found to be significantly exacerbated in head-protected rats when compared to hind limbs-protected ones, as evidenced by both qualitative histological analysis and histological scoring. Indeed significant differences in both structural and cellular alterations of organs were found when comparing head- and hind limbs-protected rats. Liver structure of head-protected rats was more altered than in the case of hind limbs-protected rats, with marked disorganization of hepatocyte rows and sinusoidal dilatation. Glomeruli/tubular structures and epithelial cells were also more altered in the kidney of head-protected rats. Moreover deeper lesions (up to the medulla) were observed in head-protected rats only. In ileum, irradiation induced villi shortening in both irradiated rat groups, as previously reported [24,25], but crypt and chorion structures, as well as enterocytes and their brush border were found to be more altered in head-protected rats. Overall, fibrosis was only detected in liver and kidney of head-protected rats, while it was absent in hind limbs-protected rats. Necrosis was increased in head-protected rats for all organs investigated in this study. Vascular alterations (liver sinusoids, ileum capillaries, kidney vessels) were also more obvious in head-protected rats when compared to hind limbs-protected rats. Therefore irradiation induced disorganisation of liver, kidney and ileum structures in both groups of irradiated rats but structural and cellular alterations were found to be more significant when rat head was protected from irradiation.

Altered or delayed hematopoietic recovery in head-protected rat is unlikely to explain the differences we found regarding organ damage between the two irradiated rat groups, as we found similar kinetics of white blood cells and spleen recovery for hind limbs- and head-protected rats. Moreover all visceral organs studied (liver, kidney and ileum) were exposed to the same radiation dose (12 Gy) and were located outside of the narrow penumbra field for both hind limbs- and head-protected rats, ruling out involvement of dose distribution in the differences we found between hind limbs- and head-protected rats. Therefore our results indicate that the extent of organ damage is linked to the body territory shielded. Moreover, our data indicate that organ damage is exacerbated when the head is protected, suggesting that cranial/brain exposure may trigger mechanisms leading to visceral organ protection.

Irradiation-induced late organ damage is thought to be linked to pro-inflammatory mediator release, increased production of reactive oxygen species and damage to vessels [6–8,13–16]. Therefore differences in organ damage between our two rat models may be explained by the modulation of one or all of these pathways. Our histology data indicate that vascular alterations were exacerbated when the head was protected from irradiation, suggesting that they may contribute to the enhanced organ damage observed in case of cranial/brain protection. Histology analysis also reveals that the occurrence of inflammatory cells in ileum is slightly increased in case of head protection (Table 3). In line with this, crypt inflammation was only observed in the ileum of head-protected rats (Table 3). Altogether this suggests that an enhanced chronic inflammation may also in part explain the exacerbated organ damage observed in case of cranial/brain protection. In accordance with these observations, CXCL1 plasma levels were only found to be increased in head-protected irradiated rats for the 2 weeks preceding organ damage analysis, indicating that a late enhanced chronic inflammation may in part explain why organ damage was exacerbated in case of head protection.

In line with this, we found significant differences regarding the early inflammation status of hind limbs- and head-protected rats. Indeed, while head-protected rats still exhibited high haptoglobin level 4 days post-irradiation, plasma haptoglobin level of hind limbs-protected rats had already returned to control values, indicating that acute inflammation was significantly longer in head-protected rats. Together with the late chronic inflammation, this may contribute to the exacerbated organ damage observed in head-protected animals. In contrast, acute inflammatory reaction was shorter in case of cranial/brain irradiation. Moreover, according to CXCL1 measurements, late chronic inflammation was reduced in hind limbs-protected rats during the two weeks preceding organ damage analysis. Therefore reduced acute and chronic inflammation may in part explain why visceral organ damage of hind limbs-protected rats was attenuated when compared to head-protected animals. The shorter inflammatory reaction observed after head exposure may be linked to the higher plasma level of the anti-inflammatory hormone corticosterone we measured in hind limbs-protected rats 4 days post-irradiation. In accordance with these hypothesis, corticosteroid administration has been proved to be efficient to prevent or delay lung pneumonitis and kidney nephropathy [10,11].

The early corticosterone increases we measured in irradiated rats indicate that irradiation triggers activation of the Hypothalamic-Pituitary-Adrenal (HPA) axis, whether the head is exposed or not, which is in accordance with previous reports [33–35]. It has been suggested that after irradiation, HPA axis activation may be linked to central inflammation caused by increased expression of pro-inflammatory cytokines such as IL-1 or TNF-α in brain regions such as the hypothalamus [18,35,36]. Increased expression of pro-inflammatory cytokine in the hypothalamus even occurs without direct brain irradiation and there are evidences indicating that the expression of pro-inflammatory mediators in the hypothalamus depends in part on ascending information (regarding peripheral inflammation) conveyed by afferent fibers of the vagus nerve [18]. Afferent fibers from the vagus nerve end in the dorsal vagal complex including the nucleus of the solitary tract, which is known to regulate hypothalamic nuclei such as the paraventricular nucleus [37]. This hypothalamic nucleus is responsible for the control of the HPA axis through the release of Corticotropin-Releasing Hormone (CRH) [18,33,37,38]. Therefore, after irradiation, information regarding peripheral inflammation may be partly conveyed to brain nuclei through the vagus nerve, resulting in stimulation of anti-inflammatory pathways that may include the HPA axis with subsequent anti-inflammatory hormone release, the cholinergic anti-inflammatory pathway (through efferent fibers of the vagus nerve) and also the sympathetic nervous system, which innervates peripheral and lymphoid organs [37,38]. As visceral organs from head- and hind limbs-protected rats were exposed to the same radiation dose, initial inflammation in visceral organs may be similar for both groups of irradiated animals and inflammatory signals may be partly conveyed to the brain through afferent fibers of the vagus nerve, as previously demonstrated [18]. However, in the case of hind limbs-protected rats, direct brain irradiation may trigger higher expression of pro-inflammatory cytokines in areas such as the hypothalamus when compared to head-protected animals, as brain irradiation is sufficient to activate the HPA axis in rats [35]. This may result in enhanced activation of anti-inflammatory pathways such as the HPA axis, the cholinergic anti-inflammatory pathway, and the sympathetic nervous system, leading to attenuated organ damage [37,38]. In accordance with this hypothesis, we measured higher corticosterone plasma levels in case of head exposure during the acute phase of irradiation, suggesting indeed that brain irradiation promotes higher activation of the HPA axis. However, based on vagotomy experiments in total-body irradiated rats, another report suggests that the vagus nerve may be a pro-inflammatory pathway in early irradiation-induced inflammation in the case of the intestine [39]. Therefore, according to this study, the attenuated ileum damage we observed in case of brain exposure could also be related to a reduced activity in the efferent fibers of the vagus nerve.

We also detected late increase (50 days after irradiation) in corticosterone plasma levels in hind limbs-protected rats only. Delayed effect of brain irradiation such as impairment of the negative feedback exerted by glucocorticoids has been described recently in rats [40]. Therefore the late corticosterone increase we report here may be related to an impairment of the negative feedback, resulting in enhanced release of adrenal corticosterone.

The late increase in corticosterone plasma levels measured in hind limbs-protected rats was timely correlated with the late body weight loss observed in these animals only, suggesting a possible link between these parameters. In contrast, the different extent of organ damage we observed in the two groups of irradiated rats seems to be independent of the late phase of body weight loss. Indeed, at the time they had developed the late phase of body weight loss (64 days post-irradiation), histological lesions were attenuated in hind limbs-protected rats when compared to head-protected ones. The explanation for this late body weight loss remains to be investigated, but it is unlikely to be linked to the reduced absorption surface of ileum we observed, as structural ileum alterations were observed in both hind limbs- and head-protected rats. Plasma albumin levels of hind limbs-protected rats were not different from those of age-matched controls between 10 and 101 days post-irradiation, suggesting that severe under-nutrition is unlikely to explain their late body weight loss [23]. These results also indicate that these animals have no signs of hemoconcentration and therefore are not dehydrated [22]. Therefore the late body weight loss observed in hind limbs-protected rats may rather be due to late hormonal/metabolic disorder linked to irradiation of head-localized structures such as the hypothalamus or salivary glands [41,42]. The initial trigger of such late occurring events may be the lack of progenitor cells to replace mature cells [43,44].

To conclude, we show here that the extent of visceral organ damage is attenuated in case of cranial/brain exposure, and also that this may be partly related to reduced acute and chronic inflammation. Therefore our results provide some evidences for a role of the central nervous system in controlling the development of late organ damage following high-dose irradiation. Further investigations are needed to precisely define how brain irradiation can influence late visceral organ damage.
